# Assessing students approaches to learning using a matrix framework in a Malaysian public university

**DOI:** 10.1186/2193-1801-3-54

**Published:** 2014-01-26

**Authors:** Hee Chong Teoh, Maria Chong Abdullah, Samsilah Roslan, Shaffe Mohd Daud

**Affiliations:** Foundation Studies Department, UPM, Serdang, 43400 Malaysia

**Keywords:** Student approaches to learning, Matrix framework of learning approaches, Malaysian universities

## Abstract

This study aims to evaluate the learning characteristics of students using a matrix framework of learning approaches (MFLA) in a Malaysian public university. A survey form based on Biggs’s study process questionnaire (SPQ) was distributed to a total of 350 students. This study employed a descriptive correlation research design to address the research objectives. The findings revealed that Malaysian students are prone to applying the achieving approach in their studies. The achieving approach is the most preferable learning characteristic. The results also indicated that four of the nine hypothetical learning approaches exist, two of which are positive in nature. As a result, a proposed teaching method based on the MFLA was introduced to suit the needs of these major learning characteristics among students.

## Introduction

How learners process and handle information will determine student approaches to learning (SAL), and subsequently affect the quality of their learning outcomes. According to Marton and Säljö (1976), there are two different ways of learning “surface” and “deep” approaches. Students adopting a surface approach only aim to achieve the minimum requirements, whereas deep learners will study detailed content precisely, aiming for complete comprehension of the meaning. Biggs (1987) added an “achieving” approach, that is, where learners focus on obtaining high grades in their study. These three SAL will be the main focus of the discussion pertaining to learning processes in this study.

The issue of SAL has drawn the attention of academics due to its close relationship with the academic achievement of students (Biggs and Moore, 1993; Goh et al., 2012; Kek et al., 2007). These studies reveal that a surface approach to learning is related to poor quality processes and outcomes, whereas a deep approach to learning is related to high quality processes and outcomes. The achieving approach also tends to do well in exams, but is more externally driven to gain higher grades (Biggs and Moore, 1993). Goh et al. (2012) conducted a study of SAL in Malaysian public higher learning institutions and revealed the same findings.

Notwithstanding previous findings which support the importance of deep approach in learning processes, many scholars tend to perceive Asian or South East Asian students as “surface” learners. They rely very much on the syllabus and textbooks, and are more teacher-directed and less self-directed in classroom discussion (Kember, 2000; Leung et al., 2007; Tani, 2005; Ziguras, 2001). In Hong Kong, scholars have criticized construction students for their tendency to adopt a surface approach due to their pragmatic attitude, and their eagerness for quick and instant benefits (Leung et al., 2006). Ziguras (2001), after visiting five transnational institutions located in Malaysia, Vietnam and Singapore, concluded that students in these institutions were of a “spoon-fed” type, with a lack of self-directed learning, and a desire for close supervision from teaching staff.

In the Malaysia context, Fung (2010) studied Malaysian secondary and undergraduate students, describing them as surface rote learners, unfamiliar with deep approaches to learning. The aim of their study was merely to pass examinations and to get a good job after graduation. Thang and Alias (2007), and Thang (2009) also revealed that the majority of students from public and private universities in Malaysia lacked personal autonomy and preferred a teacher-centred approach to learning. Ziguras (2001), quoting lecturers’ feedback indicated that Malaysian students expected to be spoon-fed, were scared of saying the wrong things, and wanted more direction, supervision and greater attention from lecturers. Both studies are in agreement with previous studies where researchers have criticized Malaysian undergraduates as accustomed to the traditional method of teaching, as dependent, and as adoptive of a surface and reproductive approach to learning (Ali, 2000; Smith, 2001).

However, Ling et al. (2005), while comparing approaches to learning between Australian and Malaysian undergraduates in private educational institutions, found that there was no significant difference in the deep approach. Students in Malaysia scored slightly higher in the surface approach, but the magnitude was very small. Thang (2005), in research conducted on distance learners and campus learners at one of the public universities in Malaysia, also revealed that the score for the deep approach was higher irrespective of learning modes. These findings were also confirmed by Malakolunthu and Joshua (2012).

Therefore, this study will further examine the phenomenon of SAL in Malaysia and will deepen the understanding of this learning characteristic using the matrix framework. The matrix framework of learning approaches (MFLA) was first introduced by Leung et al. (2004). The MFLA consists of nine cells which include a combination of three learning motives (LM) and three learning strategies (LS). By recognizing the learning characteristics of undergraduates, this study aims to help students and teachers to identify their weaknesses and to find solutions for the difficulties faced.

## Matrix framework of learning approaches (MFLA)

This discussion of MFLA is an extension of Biggs’s learning classification of three learning approaches; the deep approach, surface approach and achieving approach. Biggs’s classification is derived from LM and LS. In other words, each approach can be explained from the dimensions of motive and strategy as shown in Table 1.

**Table 1:** **Approaches to learning (motives/strategies)**

Learning	Surface	Deep	Achieving
Motive (LM)	Reinforced by punishment or rewards.	Intrinsic interest, thirst for knowledge.	Achievement, high grades and winning prizes.
Strategy (LS)	Rote learning, narrow targets, reproduce knowledge.	Maximize meaning, high cognitive level.	Effective use of space and time.

Source: Biggs et al. (2001).

The LM for surface approach is extrinsic. An individual who carry out the task using this approach is normally afraid of failure, has no intention to excel in his work and simply want to complete the task requirements (Biggs and Moore, 1993; Entwistle, 1987). In Malaysia, this phenomenon is so obvious among Chinese undergraduate with the manifestation of *kiasu-ism*, which means “afraid of losing out” or “lose of face” (Goh, 2006). Whereas the LS for surface approach refers to students obtaining information in a random pattern for short-term recall and never challenge the validity of the information and deploy the rote memorization strategy (Hashim, 2009; Jewels and Ford, 2004). However, there are scholars who argued that there are differences between memorizing with understanding and memorizing without understanding (Dahlin and Regmi, 1995; Dahlin and Watkins, 1997; Meyer and Shanahan, 2003). This distinction is believed to resolved the ‘paradox of Chinese learner’ which referred by Marton et al. (1993). Nevertheless, the various contrasting form of memorizing and repetition in learning processes is not within the scope of current discussion.

The LM behind deep approach is intrinsic and the intention is to understand the content of reading texts (Entwistle, 1987). Learners who apply this approach assume learning is personal commitment, which means that they seeks the knowledge with interest and curiosity, related the content to previous knowledge and experience, abstract thinker (Arteche et al., 2009; Biggs and Moore, 1993; Entwistle, 1987), with high academic expectation (Rodriguez, 2009) and altruistic life goals (Wilding and Andrews, 2006). Various LS used in deep approach such as possess wide knowledge relate to content, combining a variety of resources, discussion of ideas with others, reflect metacognitively on study, enjoy the process, be prepared to invest time and effort, and applying knowledge in real world situations (Biggs and Moore, 1993; Nelson Laird et al., 2008). Students adopting deep approach tend to focus on the writer’s intention and their main aim is to develop an understanding of what they are reading.

The LM of achieving approach is the need for achievement, with the intention to obtain as higher mark as possible (Entwistle, 1987). It usually relates to the outcomes based on competition and ego enhancement (Leung et al., 2007). Unlike deep approach, the involvement of students in their tasks is not an end itself, but as the means to obtain higher grades (Biggs and Moore, 1993). The LS of achieving approach may consist of organizational study method, competing with others, precise calculation and goal-directed. Study methods adopted by students who using this approach are very “technical” such as keeping clear and neatly notes, optimal use of time, planning in advance and highly self-discipline (Biggs and Moore, 1993).

However, according to Leung et al. (2004, p. 190), “various learning phenomena encountered could not be explained by these three learning approaches in isolation”. Sometimes students may not be interested in the program, but a good lecturer can contribute to leading or guiding them towards a better learning strategy such as achieving, whereas a heavy workload or a factual type of examination format may influence deep learners to apply a surface strategy to survive studying. This phenomenon was demonstrated in Leung et al. (2004), where it was revealed that the majority of Hong Kong construction engineering students adopted the surface-achieving learning approach. Leung et al. (2004) named these nine cells by characterizing them into nine different learning characteristics (Table 2).

**Table 2:** **Matrix framework of learning approaches**

	Surface motive (SM)	Deep Motive (DM)	Achieving Motive (AM)
Surface Strategy (SS)	***Surface Approach (SA)***	***Discouragement Approach (DCA)***	***Avoid Failure Approach (AFA)***
	*Not interested in the subject so he/she is not willing to spend time on it.*	*Interested in the subject but his/her interest is discouraged by the learning environment, such as an overcrowded timetable.*	*(Low need-achiever) Is afraid of failure. Likes tasks with high success rates to gain feelings of self efficacy. Therefore, he/she is interested in easy tasks.*
Deep Strategy (DS)	***Encouragement Approach (ECA)***	**Deep Approach (DA)**	**Achieve Success Approach (ASA)**
	*Not interested in the subject but a contribution is made because of a good learning environment.*	Intrinsically interested in the subject so he/she is willing to spend extra time on reading related material.	(High need-achiever) Is very aggressive and has a strong mind to win. The more difficult the task, the greater the glory. Therefore, a competitive challenge is the only factor to motivate him/her.
Achieving Strategy (AS)	***Fear of Failure Approach (FFA)***	**Hardworking Approach (HWA)**	**Achieving Approach (AA)**
	*Not intrinsically interested in the subject but fears getting a bad academic result and so he/she works hard.*	Interested in the subject and is a hardworking student.	Has the characteristics of both the high need-achiever and the low need-achiever, but no matter which strategy is used, getting a high mark is the end purpose.

Source: Leung et al. (2004).

Labels in italic represent negative learning approaches.

Referring to Table 2, the column with italic boldface label represents negative approaches in the MFLA. There are the surface approach (SS-SM), discouragement approach (SS-DM), avoid failure approach (SS-AM), encouragement approach (DS-SM) and fear of failure approach (isme AS-SM). These approaches consist of some weaknesses which are either caused by the personality of learners, such as a lack of interest, low self-esteem, high defensiveness and a fear of failure or *kiasu-*(Goh, 2006), or because of the an unfavorable learning environment, for example the assignment is too difficult and the teacher’s expectation is too high (Leung et al., 2004). The identification of these learning approaches can help students and teachers to eradicate such discouraging factors and aim towards positive approaches in learning.

There are four positive approaches in the MFLA, namely the deep approach (DS-DM), achieve success approach (DS-AM), hardworking approach (AS-DM) and achieving approach (AS-AM). Basically, the learners who fall into this group are high need achievers with a high interest in learning and are intrinsically motivated to achieve success in their study. According to Leung et al. (2004), training in metacognitive study skills will further enable this group of students to achieve excellent performance.

## Objective

This study aims to evaluate the learning characteristics of students in a Malaysian public university using a matrix framework of learning approaches (MFLA). Specifically, there are two objectives to achieve in this study namely to identify the student approaches to learning (SAL) based on the traditional learning approaches, and on learning motive (LM) and learning strategy (LS). Two research questions were formulated to guide the following discussion.

1. What was the preferred SAL among students in a Malaysian public university?
2. What were the combinations of LM and LS found among students in a Malaysian public university?

## Methodology

### Participants

This study employed a descriptive-correlation research design with a questionnaire survey. A cluster or multistage sampling method was used to identify the samples of a total of 350 undergraduates in University Putra Malaysia (UPM), one of the research universities in Malaysia, to participate in the study (Cerin, 2010; Creswell, 2008).

According to Creswell (2008), this sampling method is appropriate when the population involved in research is large or difficult to identify. In this study, the accessible population is approximately 14,000 which are separated into 78 programs offered in UPM’s main campus, located at Serdang Malaysia (information updated to May, 2013). Researchers regarded each of this program as a primary cluster, therefore, 15 percent of these clusters i.e. 12 programs, will be randomly selected as to represent the whole population (Dalen, 1973, p. 323). Thus, by using the fish bowl technique, researchers have chosen 14 programs with an extra two programs as a precautionary step against the possibility of incomplete data or failure to obtaining approval from the relevant party.

Following the random selection of the 14 programs offered by the university, the respondents from the second year or semester 4 in this program were randomly selected to represent the cluster. There are two reasons why second year (semester 4) students were selected as samples in this study. Firstly, second year students have had a wider range of experiences during university life and can arguably provide more accurate assessments about a variety of university activities (Tinto, 2000). Secondly, third year students (also final year students in some faculties) are doing their final year project, or attending industrial training, or practicum which are not available in campus and has no classroom context to evaluate.

Researchers randomly selected the core subject for the second year students in each program to assess the feedback because respondents in the same cluster are required to evaluate the same teaching and learning context. The rationale behind this selection is not only because classrooms served as logical units of analysis, but also because this procedure greatly simplified the task of reaching students (Tinto, 1997). Hence, the core subject chosen is regarded as the secondary cluster.

Finally, respondents from the selected core subject were randomly selected to form the final sample of study. This combination of cluster random sampling with individual random sampling is recommended by Fraenkel and Wallen (2012, p. 97). Researchers applied the non-proportionate quota sampling method for individual sampling so as to guarantee a minimum sample size from each secondary cluster. With the specify number of respondents from each cluster, it avoided the small sample size from the classes which reduce the chance in selecting non-typical respondent biasing the results.

By going through this sampling procedure, the wide coverage of the respondents is expected to reach and ensure the representation of the sample to the targeted population. The students will complete the questionnaire distributed to them. In order to secure responses, the questionnaire was administered during the class session, and the return rate was 100%.

### Measurement

In this study, the existing SPQ designed by Biggs (1987) was adapted and used to collect the research data from respondents. Study Processes Questionnaire, SPQ (Biggs, 1987) was developed to reflect the findings of both quantitative and qualitative research into how students study (Biggs, 1987). Both research paradigms have confirmed the two most basic approaches that students tend to utilize which were first identified in qualitative research by Marton and Säljö (1976). As discussed earlier, students who are learning because of extrinsic motivational factors or fear of failure tend to adopt superficial strategies, and students who are interested in what they are studying are likely to adopt strategies, which help their understanding of the material. These contrasting ways of studying are known as the “surface” and the “deep” approach, respectively. While students tend to be relatively consistent in terms of which of these approaches they adopted, they also modify their approach depending on their perceptions of course requirements and other factors (Biggs, 1987; Entwistle and Ramsden, 1983).

The modified SPQ in this study contains 27 items divided among the three approaches to learning (deep, surface and achieving) into six motive and strategy scales. Each response to an item was to be answered on a four point Likert scale that described the match with the respondent’s behaviour: 1 = strongly disagree and 4 = strongly agree, respectively. The scores on each SAL will be added to form summation scores. For data description purpose, researchers created the class interval as follow to represent the data distribution. The three classes created will be named as high level, moderate level and low level on surface approach, deep approach and achieving approach (Table 3).

**Table 3:** **Level and class interval for SA, DA and AA**

Level	Class interval width	Class	Class boundary		The next class lower class boundary (LCB)	Class interval (exact limit)
			Lower Class Boundary (LCB)	Upper Class Boundary (UCB)		
Low	1	1.00–2.00	1.00	2.00	2.01	1.00–2.00
Moderate	1	2.00–3.00	2.01	2.99	3.00	2.01–2.99
High	1	3.00–4.00	3.00	4.00	-	3.00–4.00

Note: SA = surface approach; DA = deep approach; AA = achieving approach.

The Cronbach’s alpha for the surface, deep and achieving approach are shown in Table 4. According to Ary et al. (2008, p. 249), “a good reliability is one that is as good as or better than the reliability of competing measures”, and Tan (2005, p. 137) has reported that most studies employing SPQ show internal consistency reliability exceeding 0.5, therefore, the current alpha value exceeding 0.7 was considered satisfactory.

**Table 4:** **Cronbachs’ alpha for SAL**

Concept		Items	Alpha value
SAL			
	Surface approach	8	0.73
	Deep approach	9	0.81
	Achieving approach	10	0.78

Descriptive statistics were used to measure mean and standard deviation for exploration of the variables. As to answer both research questions, the correlation coefficients and cluster analysis were used in this study. Correlation coefficient is a statistical method to measure correlation among variables. The sign of a correlation coefficient (+ or -) indicates the direction of relationship between two variables. Besides, a correlation coefficient ranges from ±1–0 indicates the strength of relationships.

Whereas, cluster analysis allowed researcher to make grouping base on objects or distance (Hair et al., 2010). The patterns of relationships among surface, deep and achieving approaches were examined using non-hierarchical *k*-means cluster analysis. K-means cluster analysis has proved effective in minimizing differences within, and maximizing differences between, the clusters (Meyer and Shanahan, 2003).

## Findings

### What was the preferred SAL among students in a Malaysian public university?

To address the above research question of this study, the data obtained was first analyzed using descriptive statistics. Findings in Table 5 indicate that the learning approach most preferred by students was the “achieving approach” (*M* = 3.07), followed by the “deep approach” (*M* = 2.94) and the “surface approach” (*M* = 2.28). Based on the level assigned to the mean scores in Table 3, the three learning approaches among students were distributed to high level for achieving approach, moderate high level for deep approach and moderate low level for surface approach.

**Table 5:** **Distribution of students learning approaches**

Descriptive statistics	Variables		
	SA	DA	AA
Mean	2.28	2.94	3.07
Standard deviation	0.47	0.43	0.41

Note: N = 350; SA = surface approach; DA = deep approach; AA = achieving approach.

The three learning approaches in Table 5 were further analysed using *k*-means. The surface, deep and achieving approach scores converted to range −1 to +1. The cluster analyses of these three learning approaches scales were conducted sequentially from two clusters onward until a stable (or nearly so) cluster membership across two successive cluster solutions reached (Meyer and Shanahan, 2003; Quinn, 2011).

**Figure 1:**
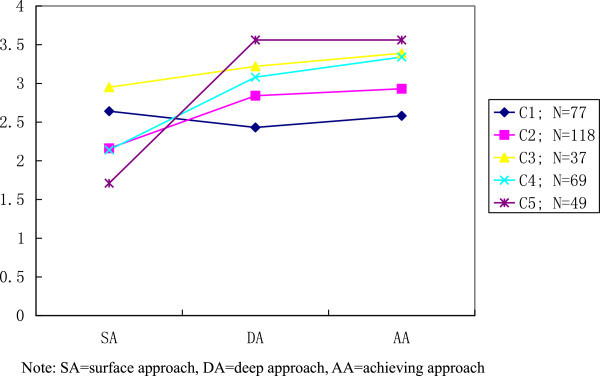
**Five-cluster solution for student approaches to learning.**

**Figure 2:**
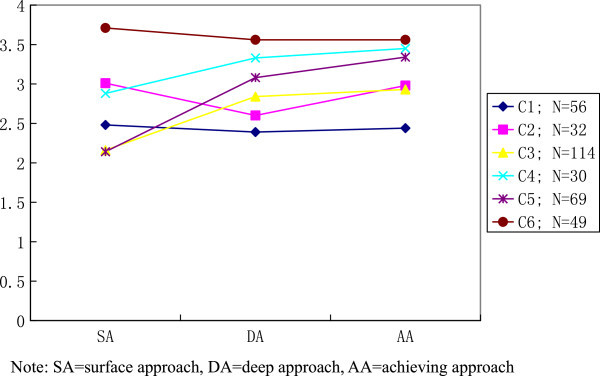
**Six-cluster solution for student approaches to learning.**

Results from the *k*-means cluster analyses for the traditional learning approaches are shown in Figures 1 and 2. These two figures show the different clusters of respondents in two successive solutions, and also the cluster-mean scores for the three learning approach variables, namely surface, deep and achieving approaches. The five-cluster solution is considered first. Apparently, all clusters exhibited in Figure 1 show relatively higher level of achieving approach, except cluster 1 and 5. Cluster 1 (about 22% of the respondents) demonstrated with moderate high surface approach, followed by achieving and deep approach. Meanwhile, 49 respondents in Cluster 5 (14% from the sample) exhibited similar mean scores for both deep and achieving approach. Be that as it may, majority of respondents in Cluster 2–4 (64%) have shown preference adopting achieving approach in their study, which is in agreement with the finding in Table 5.

The six-cluster solution presented in Figure 2 depicted cluster features identical to those in five-cluster solution (in Figure 1). Approximately 61% of respondents (Cluster 3–5) exhibited preference of adopting achieving approach in their learning process. These three clusters are similar with Cluster 2–4 exhibited in Figure 1 with highest mean score for achieving approach, followed by deep and surface approach. Whereas, Cluster 1, 2 and 6 have shown the relative higher mean scores in surface approach compared with the other two learning approaches. Both Cluster 1 and 6 have shown a very flat profile consisting very similar mean scores for all learning approaches. Meanwhile, Cluster 2 of the six-cluster solution is similar to Cluster 1 in Figure 1 with the lowest means for deep approach.

### What were the combinations of LM and LS found among students in a Malaysian public university?

Table 6 highlights in bold the preferred scores regarding LM and LS. The findings indicated that “achieving motive” and “achieving strategy” were the most popular learning approaches among students in Malaysia. This is in compatible with the findings of SAL used in the traditional way where the “achieving approach” was the most popular learning approach (refer to Table 5). According to Leung et al. (2004, p. 192), students who preferred an “achieving approach” are those students who “has the characteristics of both the high need-achiever and the low need-achiever, but no matter which strategy is used, getting a high mark is the end purpose”. However, among the six mean-scores presented in Table 6, only achieving motive has reached the interval of high level (refer to Table 3), whereas the other LM and LS fall in the moderate levels.

**Table 6:** **Scores of Learning Motives (LM) and Learning Strategies (LS) among students**

	Learning motives			Learning strategies		
	Surface motive	Deep motive	Achieving motive	Surface strategy	Deep strategy	Achieving strategy
**Mean**	2.15	2.99	**3.24**	2.42	2.87	**2.90**
**SD**	.56	.44	.44	.49	.50	.50

The preferred LM and LS is highlighted in boldface.

Table 7 shows significant correlations between most LM and LS. This study reconfirms traditional learning phenomena; for instance, SM leads to SS (.631 at a significance level of .0001), DM is strongly related to DS (.703 at a significance level of .0001) and AM induces AS (.519 at a significance level of .0001). Besides, there are three cells with negative correlation, namely the correlation between SS and DM (−.17 at a significance level of .001), SM and DS (−.33 at a significance level of .0001), and SM and AS (−.28 at a significance level of .0001) indicate the existence of both motive and strategy are inverse. These results are in agreement with the fundamental principle of learning approaches, the surface and deep are mutually exclusive and no students would maintain both approaches simultaneously (Biggs and Moore, 1993; Leung et al., 2004).

**Table 7:** **Pearson correlation of learning approaches**

		SM	DM	AM
SS		.63^**^	-.17^**^	.06
	Sig.	.00	.00	.24
DS		-.33^**^	.70^**^	.43^**^
	Sig.	.00	.00	.00
AS		-.28^**^	.69^**^	.52^**^
	Sig.	.00	.00	.00

Note: SM = surface motive; DM = deep motive; AM = achieving motive; SS = surface strategy; DS = deep strategy; AS = achieving strategy.

**Correlation is significant at 0.001 level (2-tailed).

In addition, Table 7 shows a clear indication that DM significantly induced AA (.69 at significance level .0001), but it happened reversely for SM and AS (−.28 at significance level .0001). According to Biggs and Moore (1993, p. 314), an achieving approach may be linked to either surface or deep approach, “One can rote-learn in an organized or an unorganized way, or seek meaning in an organized or unorganized way”. In this study, the Pearson correlation on the variables revealed that the students in Malaysia show the apparent tendency in deep-achieving approach. Nevertheless, the pattern of relationships between the motive and strategy among respondents need to be further investigated with *k*-means cluster analysis.

**Figure 3:**
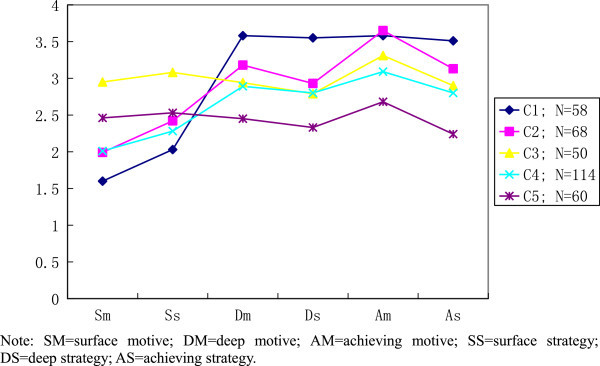
**Five-cluster solution for LM and LS learning approaches.**

**Figure 4:**
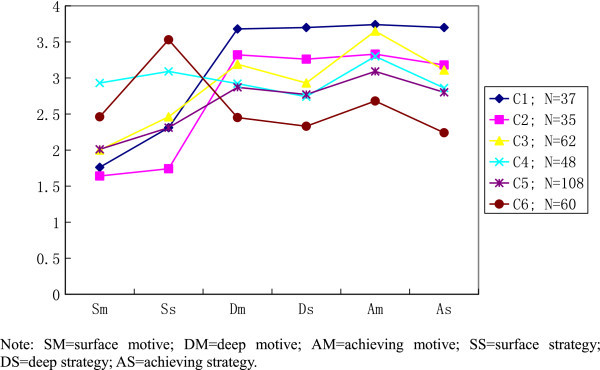
**Six-cluster solution for LM and LS learning approaches.**

Results from the *k*-means cluster analyses are shown in Figures 3 and 4. The five and six cluster solution can be discussed in three major groups with many similar respects. The first group is Cluster 1 (about 16.6% of respondents) in Figure 3 and Cluster 1 and 2 (about 20% of respondents) in Figure 4. The Cluster features in this group are exhibited with high deep and achieving cluster-means, which are nearly flat for their profile features. Contrary, the respondents in this group reported a low surface approach (both motive and strategy).

The second major group comprises of Cluster 2 and 4 (about 52% of respondents) in Figure 3, and Cluster 3 and 5 (about 49% of respondents) in Figure 4. Obviously, this is the large proportion of respondents which seem to study with high achieving motive. Unlike group 1, this group of students has adopted relatively lower deep approach to their learning. Identical to previous group, this group also demonstrated a relatively low surface cluster-means.

The third group is more likely to consist of students who prefer mixed learning approaches. Cluster 3 and 5 (about 31% of respondents) in Figure 3, and Cluster 4 (about 14%) in Figure 4 with very similar mean scores on all three learning approaches. This group of students has not shown their distinct preference on any of the learning motives or strategies. In addition, the diverse proportion shown in this group for Figures 3 and 4 is due to an additional cluster (Cluster 6, about 17% of respondents), showing extremely high cluster-mean score on surface strategy. After observing the profile of the respondents in this group, they seem to have adopted avoid failure approach (achieving motive and surface strategy) which is a typical characteristic of low need-achiever.

As to recap the combination of Pearson Coefficients and Cluster Analyses, apparently the three traditional learning approaches for surface, deep and achieving does exist in this study. However, the other proposed learning approaches in MFLA are not clearly exhibited, especially through the cluster analyses. In other words, besides the traditional learning approaches with similar motive and strategy, the cross-over LM and LS learning approaches are not clearly emerged in current study.

## Discussion

The results of the current study, based on Pearson coefficients and Cluster analyses, revealed that most of the students preferred an “achieving approach” rather than a “deep approach” or a “surface approach”. This finding deviates from previous studies which argue that Malaysian students are surface learners (Fung, 2010; Ling et al., 2005; Ali, 2000) or deep learners (Thang, 2005). As Biggs et al. (2001) have emphasized that learning approaches are the outcome of both individual characteristics and the teaching context; therefore, this result should be read in accordance with the nature of the relationship between the teaching context, students and task.

Furthermore, we should cautious with the high scoring level on the “achieving approach” because it does not indicate that we have progressed much further than the “surface approach”. According to Biggs and Moore (1993), “the achieving approach is like the surface approach in that it is focused on the product”. The difference of achieving approach compared with surface approach is that the former focuses more on obtaining high grades and winning prizes rather than avoiding failure. This is not surprising due to the examination oriented culture that has long existed in the Malaysian educational context (Yoong, 2009). Tan (2005) also commented that Malaysian adult learners are more substantially influenced by “face value”, which means that they are more driven by external factors such as social status and economic achievement.

Unlike the other two learning approaches, achieving approach is more focused on how learners organize their time and technique to engage with the task (Biggs and Moore, 1993). This approach is the means and not the end itself. Hence, students’ work depends on what earns the most marks. In other words, if a teacher requests students to relate content knowledge into a real life context or operate at a high, or abstract level of conceptualisation, the achieving approach will associate more with the deep approach, whereas, if a teacher rewards for the recalling of details, students will develop rote learning ability (Biggs et al., 2001). In order to clear doubt appeared in this regard, cluster analyses have provided us with overall picture of the learning characteristics of students. Apparently, majority of students have shown the relatively higher tendency in adopting achieving approach associate with deep approach instead of surface approach.

There are several reasons which determine why students who have adopted achieving approach to associate more with deep approach rather than surface approach. Teaching practices such as full of imagination and enthusiasm, energetic or prompt feedback of student work, and the issues of teacher fairness and professionalism will induce deep approach. Course design where content, delivery, activities, and assessment are all aligned to attain higher learning outcomes may also encourage undergraduates to adopt deep approach in the learning processes. Besides, when students perceive the new learning environment to be positive in terms of the clarity of its goals, the usefulness of the textbook, and the workload is manageable, it will cause them to adopt deep learning (Goh, 2008; Nijhuis et al., 2005; Pang et al., 2009).

Be that as it may, a further study on LM and LS revealed that “achieving motive” induces “achieving strategy” and “deep strategy”, but not “surface strategy”. The most popular combination of LM and LS is “AM-AS”. Therefore, a more accurate and specific term describing the learning approaches applied by the respondents in this study is achieving approach (Biggs and Moore, 1993; Leung et al., 2004). Biggs and Moore (1993) described achieving approach as identical to deep approach which involves a high degree of metalearning. Leung et al. (2004) also characterized these students as either high need-achievers or low need-achievers both aim for getting a better grade.

The cluster analyses have revealed a small proportion of students with the combination of achieving motive and surface strategy (SS-AM). According to Leung et al. (2004), this group of students has the tendency to avoid failure because they afraid of losing face. This scenario normally occurs when teacher reward the recall of details instead of comprehension of materials. The students will produce long and thorough essay but may not answering the question. They will overload lecturer’s reading and evaluation capacity until feel obliged to reward them with high grades.

The LM and LS combinations stated in Table 8 show the overall students’ characteristic’s in learning in this study. No obvious combinations of LM and LS found besides the traditional learning approaches. The suggested teaching methods to cater for these characteristics can refer to the learning strategies proposed in Table 9.

**Table 8:** **Learning characteristics of students**

	SM	DM	AM
SS	Surface approach		Avoid failure approach
DS		Deep approach	
AS			Achieving approach

Note: SM = surface motive; DM = deep motive; AM = achieving motive; SS = surface strategy; DS = deep strategy; AS = achieving strategy.

**Table 9:** **Proposed teaching methods in the matrix framework of learning approaches**

	Surface Motive (SM)	Deep Motive (DM)	Achieving Motive (AM)
Surface Strategy (SS)	**Surface Approach (SA)**	**Discouragement Approach (DCA)**	**Avoid Failure Approach (AFA)**
	Extrinsic Motivation.	Sufficient study period	Mastery learning
	Warm classroom climate.	Discussion and inductive teaching	Some techniques
	Involvement of tasks		
Deep Strategy (DS)	**Encouragement Approach (ECA)**	**Deep Approach (DA)**	**Achieve Success Approach (ASA)**
	Ownership of task	Metacognitive learning	Increasing the task difficulties
			Metacognitive learning
Achieving Strategy (AS)	**Fear of Failure Approach (FFA)**	**Hardworking Approach (HWA)**	**Achieving Approach (AA)**
	Ownership of task	Metacognitive learning	Metacognitive learning
	Expectation of success/failure		
	Organized, well structured		

Source: Leung et al. (2004).

Apparently, lecturers play a very important role in affecting learning approaches. Lecturers should have always reflected on the use of various teaching methods which are more student-focused rather than teacher-focused. As a good lecturer, one should not just do well in teaching, but must be able to be effective in pedagogical skills, classroom management, and understand the characteristic of the learner. Most importantly, a good lecturer should always be prepared to listen to the voice of students.

One-way teaching is no longer suitable for the Net-Geners nowadays. Lecturer is recommended to increase multi-way teaching approach by group discussions, practical training, project-based or problem-based learning, as well as hands-on activities in order to promote students’ study interests (Leung et al., 2007).

Lecturers should make an effort to provide prompt, detailed, and personalized feedback to the students. Students learn well when the strengths and weaknesses of their assignments, products, and performances are evaluated in detail, and they receive specific suggestions on how to improve their work. Another way to improve the assessment efficiency is to spell out performance criteria and the learning groups evaluated on each other’s work (Chickering and Kuh, 2005). This practice not only helps students to cultivate their critical thinking, but also helps to sustain motivation, excitement, and engagement of the students in their studies. Furthermore, this extensive feedback from peers, tutors, and lecturers will help students to produce high quality work instead of perceive it as a “paper” to secure for future job.

Besides that, according to Chalmers and Fuller (1996), “metacognition” refers to two aspects of thinking: awareness about cognition and control of cognition. In this study, apparently students have awareness about cognition, but what they may lack is the control or regulation of cognition. In other words, by just being aware of the knowledge is insufficient, as students have to know how to put the knowledge into practice.

There are three general processes to regulate the use of metacognitive learning: planning, monitoring and self-regulation or checking. Planning activities are normally undertaken before the beginning of a learning process. Learners have to decide which strategies to use and how to process information effectively. As a deep learner, the strategies to approach the knowledge will normally be,

- study with a intention to understand but not just for examination;
- focus on “what is signified” rather than on unrelated parts of the task;
- relate previous knowledge to new knowledge;
- relate theoretical ideas to daily experience;
- relate and distinguish evidence and argument;
- organize and structure content into a integral and holistic idea or concept.

Monitoring activities play an important role in regulating the knowledge. Learners are going through trial and error, revising, and rescheduling while applying the metacognitive skills in the learning process. Monitoring includes self-test about the comprehension of information, reflection, discussion, presentation and application to integrate and internalize the knowledge learned to become part of their lives.

Lastly, self-regulation refers to evaluation on the efficiency and effectiveness of the learning strategies used. Learners will regularly check on their own progression, performance, time management, resource management to ensure the knowledge acquired has been fully explored. These self-regulating activities are essential to ensure learners are able to adjust and correct their own mistakes from time to time.

## Conclusion and suggestions

This study examines the learning characteristics of students in a Malaysian public university. Based on the above discussions, a learning phenomenon among students in Malaysia is formulated (Table 8) using the MFLA. Four of the nine learning approaches in this MFLA were identified, namely the Surface Approach (SA), Deep Approach (DA), Achieving Approach (AA), and Avoid Failure Approach (AFA).

The results of the empirical study revealed that “achieving motive” and “achieving strategy” were the most popular LM and LS of students in the Malaysian public university. Data analysis revealed that two out of four learning characteristics adopted by students were positive in nature. Nevertheless, there were also two negative learning approaches, namely the “surface approach (SA)” and “Avoid Failure Approach (AFA)” which were presented among students with the lowest mean.

Therefore, the teaching methods of metacognitive learning are the most appropriate for application by these students. To be specific, a warm classroom environment, engagement of tasks, mastery learning and metacognitive learning should be introduced.

The sample and institution involved in this study were rather limited in size, therefore, the assessment of learning characteristics based on the MFLA should be applied to a large pool of students and to different types of higher institutions in Malaysia. The findings from such studies may enhance the understanding of the learning characteristics and help to identify suitable teaching methods for students.
